# Early morning urine collection to improve urinary lateral flow LAM assay sensitivity in hospitalised patients with HIV-TB co-infection

**DOI:** 10.1186/s12879-017-2313-0

**Published:** 2017-05-12

**Authors:** Phindile Gina, Philippa J. Randall, Tapuwa E. Muchinga, Anil Pooran, Richard Meldau, Jonny G. Peter, Keertan Dheda

**Affiliations:** 1 Lung Infection and Immunity Unit, Division of Pulmonology and UCT Lung Institute, Department of Medicine, University of Cape Town, Cape Town, South Africa; 2 Institute of Infectious Diseases and Molecular Medicine, University of Cape Town, Cape Town, South Africa

**Keywords:** TB HIV co-infection, Determine® lateral flow lipoarabinomannan assay (LF-LAM), Early morning urine (EMU)

## Abstract

**Background:**

Urine LAM testing has been approved by the WHO for use in hospitalised patients with advanced immunosuppression. However, sensitivity remains suboptimal. We therefore examined the incremental diagnostic sensitivity of early morning urine (EMU) versus random urine sampling using the Determine® lateral flow lipoarabinomannan assay (LF-LAM) in HIV-TB co-infected patients.

**Methods:**

Consenting HIV-infected inpatients, screened as part of a larger prospective randomized controlled trial, that were treated for TB, and could donate matched random and EMU samples were included. Thus paired sample were collected from the same patient, LF-LAM was graded using the pre-January 2014, with grade 1 and 2 manufacturer-designated cut-points (the latter designated grade 1 after January 2014). Single sputum Xpert-MTB/RIF and/or TB culture positivity served as the reference standard (definite TB). Those treated for TB but not meeting this standard were designated probable TB.

**Results:**

123 HIV-infected patients commenced anti-TB treatment and provided matched random and EMU samples. 33% (41/123) and 67% (82/123) had definite and probable TB, respectively. Amongst those with definite TB LF-LAM sensitivity (95%CI), using the grade 2 cut-point, increased from 12% (5–24; 5/43) to 39% (26–54; 16/41) with random versus EMU, respectively (*p* = 0.005). Similarly, amongst probable TB, LF-LAM sensitivity increased from 10% (5–17; 8/83) to 24% (16–34; 20/82) (*p* = 0.001). LF-LAM specificity was not determined.

**Conclusion:**

This proof of concept study indicates that EMU could improve the sensitivity of LF-LAM in hospitalised TB-HIV co-infected patients. These data have implications for clinical practice.

**Electronic supplementary material:**

The online version of this article (doi:10.1186/s12879-017-2313-0) contains supplementary material, which is available to authorized users.

## Background

The HIV pandemic has fueled a resurgence of tuberculosis (TB), which is the leading cause of death in HIV-infected persons in sub-Saharan Africa [1]. Rapid initiation of TB treatment may reduce mortality in these vulnerable patients making early diagnosis an imperative [2, 3]. However, there are several hurdles to diagnosis including lack of sputum production, sputum bacillary concentrations below the detection threshold of same day diagnostic tests, and atypical clinical presentation. This is further aggravated by the high frequency of extra-pulmonary (EPTB) and disseminated forms of TB with advancing immunosuppression.

Thus, there is a urgent need for new, accurate, and rapid non-sputum-based TB diagnostics that have high sensitivity in patients with HIV/TB co-infection with advanced immunosuppression [4]. The Alere Determine®-TB LAM Ag lateral flow assay (Alere, USA; referred to as LF-LAM) that detects urine lipoarabinomannan, a *M.tb* cell wall-associated glycolipid, is the most promising and only commercially available option [5]. LAM is a point-of-care test that is simple to use, requires no instruments, provides a result in just 25 min, and is low cost (<US $3.50 per test). Importantly, it is non-sputum based and is therefore useful for patients who cannot produce sputum [6]. A LAM-guided treatment strategy has recently been shown to reduce mortality in hospitalized HIV-infected inpatients with suspected TB [1]. However, LF-LAM sensitivity remains sub-optimal (40–60% in HIV co-infected patients with a CD4 count < 100 cells/ml) [7]. Thus, strategies to improve sensitivity are urgently required.

Early morning urine (EMU) is accepted as a useful method for improving the concentrations of a number of antigens and analytes found in human urine [4, 8]. The strategy has been shown to improve the yield of urine culture for TB diagnosis [9]. Thus, we hypothesised that point-of-proof concept study by collecting early morning urine, compared to a random spot urine specimen, may be a simple, low cost, and effective strategy to improve the sensitivity of LAM in hospitalised HIV-infected patients with suspected TB.

## Methods

The study population comprised HIV-infected adults (>18 yrs.) prospectively recruited between June 2012 and February 2014 from four hospitals: New Somerset, Victoria, Mitchells Plain and Groote Schuur Hospital, in Cape Town, South Africa. HIV-infected patients were referred for screening by emergency room or hospital doctors if they were HIV-infected, older than 18 years, and suspected of having TB. They were enrolled if they: 1. provided informed consent, 2. were able to provide both random and early morning urine sample, and 3. initiated anti-TB treatment either based on clinical and radiological findings, or sputum-based smear or Xpert MTB/RIF positivity. For reasons of practicality (staff only worked during office hours on week days) and ethical conduct of the study (treatment in severely ill patients cannot be delayed) patients had invariably started anti-TB treatment. Patients were excluded if: 1. all rapid sputum-based TB diagnostics were negative and no treatment was initiated based on clinical and radiological findings, and 2. they were unable to provide matched random and early morning urine samples.

All patients enrolled provided written informed consent and had basic clinical information collected, including demographics, past history of TB, presenting symptoms and vital signs. The study was approved by the University of Cape Town Human Research Ethics Committee *(HREC REF 720/2013).*

### Tuberculosis case definitions

The reference standard for TB was single sputum sample positive liquid TB culture and/or Xpert MTB/RIF assay. Definite TB required a clinical presentation compatible with TB, initiation of anti-TB treatment by the attending clinician, with any sample *M. tb* culture or Xpert MTB/RIF positive. Probable TB required a clinical-radiological picture compatible with TB, and initiation of anti-TB treatment by the attending clinician but *M. tb* culture and/or Xpert MTB/RIF were negative. As specificity was not an objective of this study non-TB patients were not recruited. However, specificity is reported in the related randomized controlled trial [1].

### TB diagnostic sampling and testing

Consultant-led hospital-based clinicians unassociated with the study determined the timing and extent of TB diagnostic work-up, and the commencement of anti-TB treatment. Routine hospital practice included the collection, where possible, of two sputum samples in patients able to expectorate. Sputum-based reference testing was performed on admission and prior to treatment (this was either on the same-day or prior to LF-LAM testing). The local reference laboratory processed all clinical specimens collected for TB diagnosis. The Xpert MTB/RIF assay and/or culture, using MGIT 960 liquid culture system (BD Diagnostics, USA), was performed on sputum samples and Xpert MTB/RIF assay was performed according to manufacturer’s instructions [10].

### Urine sampling and LF-LAM methodology

Paired samples of EMU and random urine were collected from each patient. All patients were required to give a random urine sample (30 ml) collected in a sterile container at enrolment; a random catheter sample was collected if a urinary catheter was in-situ in bedbound patients. The patients were also given a sterile container for collecting the first EMU - first void urine of the day between 05 h00 and 07 h00. The LF-LAM was done on random samples at the bedside and additionally on matched fresh bedside EMU samples the following day. All samples were tested using the Alere Determine®-TB LAM Ag lateral flow assay (Alere, USA).

Briefly, 60 μl of urine was pipetted onto the lateral flow strip loading bay (pipettes were provided with the strips). After 25–35 min, the LF-LAM was read by two independent readers blinded to the reference test results, via the following procedure: test validity was confirmed by identifying the presence of a band in the positive control window; the intensity of the colour band (if any) in the patient window was read by comparison with the pre-January 2014 manufacturer-provided visual reference scale card (graded 0–5 depending on band intensity). Using the manufacturer-recommended grade 2 cut-point (designated grade 1 post January 2014), a band of visual intensity ≥ grade 2 in the patient window was classified as a ‘positive’ test, while the complete absence of a band (grade 0) and faint band (grade 1) was classified as a ‘negative’ test. The test was reported as invalid either if no control band was identified in the patient window or if a broken/incomplete band was seen in the patient window. Invalid tests were repeated once but thereafter LF-LAM was considered to have failed. Each test was confirmed by a second reader.

### Statistical analysis

Descriptive statistics are used for baseline demographic and clinical characteristics. Diagnostic accuracy measures included only sensitivity with the 95% confidence interval. McNemar’s test was used to compare sensitivity proportion between random and EMU samples. Predictors of an increased LF-LAM grade between random and EMU was performed, using multivariate linear regression. Data was analysed, using STATA software, version 11. The STARD criteria for all reporting and analysis were used [11].

## Results

**Fig. 1:**
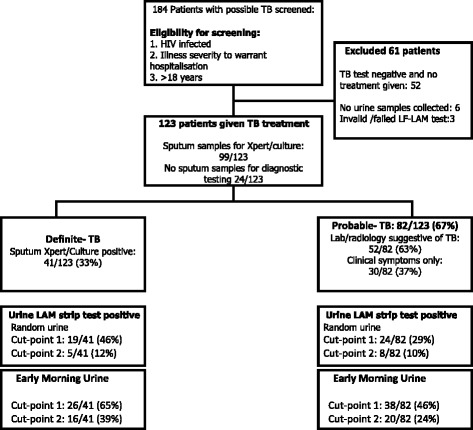
Flowchart showing study population, Xpert/culture status and diagnostic groups

184 patients were enrolled but 61 patients were excluded as they had all sputum-based TB diagnostics negative and were not given treatment (*n* = 52), or were unable to provide a urine sample at the time of enrolment (*n* = 9; Fig. 1). The median age (IQR) was 36 (31–41) years and 58% of patients were female. The majority of patients had advanced immunosuppression with a median (IQR) CD4 of 88 (36-209) cells/l (Additional file 1).

### LF-LAM sensitivity in spot versus EMU

Patient characteristics stratified by LAM strip results are shown in Table 1. 34% (41/123) of patients had culture/Xpert MTB/RIF positive definite TB from sputum, while 66% (83/123) had probable-TB with other diagnostic features suggestive of TB, including clinical findings, radiological evidence suggestive of TB (chest x-ray, ultrasound). No other biological samples were tested in this cohort. Table 1 shows the sensitivity of LF-LAM in random versus EMU samples for definite- and probable-TB groups. Overall (definite- and probable-TB combined), the sensitivity (95% CI) of EMU improved from 10% (6–17) to 30% (22–38) with EMU (*p* < 0.001) using the grade 2 cut- point. Limiting the analysis to only patients with definite TB, LF-LAM sensitivity, using the grade 2 cut-point, increased from 12% (5–24) to 39% (26–54), in random versus EMU samples, respectively (*p* = 0.005); amongst probable TB, LF-LAM sensitivity increased from 10% (5–17) to 24% (16–34) (*p* = 0.001).

**Table 1:** Overall sensitivity of the urine LAM using random versus EMU samples, and stratified by diagnostic category and preceding anti-TB treatment

Sensitivity 95% (CI)	Manufacturer recommended LF-LAM positive cut point (grade 2)		*P*-value
	Random Urine %, 95% CI, n/N	Early Morn. Urine %, 95% CI, n/N	
Definite- TB only	12 (5–24) (5/41)	39 (26–54) (16/41)	0.005
Probable TB only	9.6 (5–17) 8/82	24 (16–34) 20/82	0.001
Definite and Probable TB combined	10 (6–17) (13/123)	30 (22–38) (36/123)	<0.001
No TB Rx prior	11 (4–28) (3/27)	31 (17–50) (8/26)	0.05
TB Rx prior <7 days	10 (2–40) (1/10)	56 (27–81) (5/9)	0.04
No TB Rx prior	15 (8–27) (8/52	25 (15–38) (13/52)	0.1
TB Rx prior < 7 day	0 (0–11) (0/30)	23 (12–41) (7/30)	0.003
No TB Rx prior	14 (8–23) (11/79)	30 (18–38) (21/78)	0.03
TB Rx prior < 7 days	2 (0–12) (1/42)	30 (18–45) (12/40)	<0.001

**Fig. 2:**
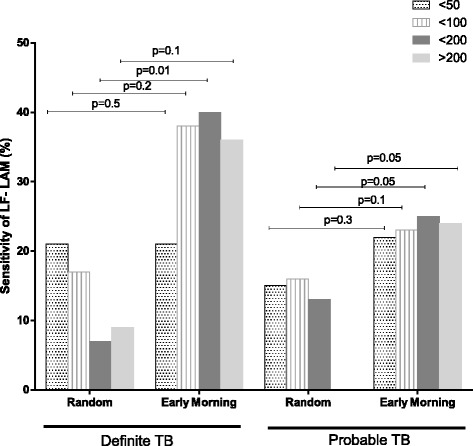
Overall sensitivity of the urine LAM strip test using random versus EMU samples and stratified by CD4 count

The data were stratified by CD4 counts (>200, <200, <100 and <50 cell/mm3) to determine whether, as in other studies, CD4 cell count played a significant role in LF-LAM grade (Fig. 2). Notably There was a significant increase in sensitivity for definite TB in the CD4 < 200 between random and EMU specimen sensitivity for individual CD4 strata (*p* = 0.01).

### Predictors of increased LF-LAM positivity between spot and EMU

None of age, Xpert MTB/RIF/liquid TB culture positivity, or prior initiation of anti-TB treatment within 7 days were significant predictors of an increasing LF-LAM grade between spot and EMU specimens (Table 2).

**Table 2:** Multivariate predictors of increasing urine LF-LAM test grade when using random versus EMU specimens

	*P*-value	Adjusted Odds Ratio	Lower 95% CI	Upper 95% CI
	No = 61			
Age, years	0.32	0.967	0.906	1.03
CD4 cell count, cells/mm^3^	0.92	1.000	0.996	1.005
TB culture/Xpert positive (pos/neg)	0.15	2.201	0.760	6.379
TB treatment < 7 days	0.41	1.59	0.53	4.81

## Discussion

The WHO has recently endorsed the use of LF-LAM testing for hospitalised HIV-infected patients with suspected TB [7]. In addition, we recently published a multicentre RCT showing that a LAM-guided early TB treatment initiation strategy reduced all-cause mortality amongst this vulnerable patient group [1]. However, test sensitivity remains sub-optimal and simple strategies that could improve test sensitivity are necessary. This proof-of-principle study suggests that using EMU can increase the sensitivity of LF-LAM testing compared to random spot urine sampling. This could further improve the utility of LF-LAM for hospitalised HIV infected patients with advanced immunosuppression and possible TB.

A number of strategies have already been published or are being examined to improve the sensitivity of LAM. These include high avidity monoclonal LAM antibodies or aptamer technologies that could improve both sensitivity and specificity [5], concentrating urine samples to improve sensitivity using centrifugation and/or molecular weight exclusion filtration, and heating to dissociate antigen and antibody [5]. These approaches improve sensitivity but has not been implemented in clinical trials. Designing a new LAM assay will take several years of development, whilst other approaches will add to cost and resource requirements. Therefore, a natural low cost method for antigen concentration, namely EMU sampling may be a practical and ease-to –implement strategy. A recent study showed no improvement in LF-LAM sensitivity from a two urine sample strategy. However, EMU sampling was performed up to seven days after the 1^st^ spot urine was collected, and the timing of morning sampling was not stipulated [12]. These methodological shortcomings may have offset the benefit of early morning sampling. Further studies are now required to confirm our findings, consider any impact on specificity, and most importantly to assess the impact on patient-important outcomes.

Our study did not find any factors that were associated with an increase in EMU LAM concentration (as measured by the change in LF-LAM grade). Factors known to affect LAM concentration, such as CD4 cell count, age and culture/Xpert MTB/RIF status were not associated with an increased LF-LAM grade [13]. This seems biologically plausible given that these factors would likely be unchanged between spot and EMU sampling time-points. It is likely that physiological factors such as blood pressure, hydration status, and consequent glomerular filtration rates are more important determinants of increased EMU LAM concentration. Interestingly, we did not find that anti-TB treatment initiated prior to spot and EMU, or between spot and EMU testing, was associated with increased EMU LAM. This does not support the “treat-to-test” hypothesis, which suggests that the concentration of bacillary antigens such as LAM may increase immediately following the initiation of anti-TB treatment due to death and metabolism of bacilli [14]. Perhaps the effect of treatment on systemic antigenemia is not as large as hypothesized and other factors such as complexing of LAM to serum proteins have a larger influence on urinary LAM concentrations.

Our study had several limitations. The sample size was small and we did not collect sufficient co-variate physiological data to adequately understand which patients are likely to have an increase in urine LAM with EMU sampling. However, resource constraints limited sample size. In many cases those with extra-pulmonary-TB had diagnosis made based on clinical, radiological findings, and did not have microbiological samples harvested from the site of presumed disease this may have led to a misclassification biased. The study did not examine the effect of EMU on test specificity and it is possible that improved sensitivity comes at the expense of decreased specificity (EMU may have increased the risk of false positive signals from bacterial or fungal cross contamination though this would seem unlikely). However, the primary aim of this study was to demonstrate proof-of-concept that EMU could improve diagnostic sensitivity; specificity of LAM is reported in the related RCT [1]. The study highlights the diagnostic implications of the timing of sample collection with EMU testing significantly improving LF-LAM sensitivity in hospitalised TB/HIV co-infected patients. Larger studies are now required to confirm our findings and importantly, to determine if the incremental sensitivity can translate to impact on patient important outcomes, or if the delay in time required between spot and EMU sampling negates the benefits of testing. In the meanwhile, it would seem sensible to collect an EMU sample for LAM testing provided treatment initiation will not be delayed.

## Conclusion

EMU could improve the sensitivity of LF-LAM in hospitalised TB-HIV co-infected patients. These data have implications for clinical practice.

## Additional file

Additional file 1: Data base incampassing all anaysed raw data. (XLSX 18 kb)

## Abbreviation

EMU: Early morning urine
HIV: Human immunodeficiency virus
LAM: Lipoarabinomannan
LF-LAM: Lateral flow lipoarabinomannan assay
M.tb: Mycobacterium tuberculosis
POC: Point-of-care
WHO: World Health Organisation
